# The Born in Bradford COVID-19 Research Study: Protocol for an adaptive mixed methods research study to gather actionable intelligence on the impact of COVID-19 on health inequalities amongst families living in Bradford

**DOI:** 10.12688/wellcomeopenres.16129.1

**Published:** 2020-08-13

**Authors:** Rosemary R C McEachan, Josie Dickerson, Sally Bridges, Maria Bryant, Christopher Cartwright, Shahid Islam, Bridget Lockyer, Aamnah Rahman, Laura Sheard, Jane West, Deborah A Lawlor, Trevor A Sheldon, John Wright, Kate E Pickett, on behalf of the Bradford Institute for Health Research COVID-19 Scientific Advisory Group

**Affiliations:** 1 Bradford Institute for Health Research, Bradford Teaching Hospitals NHS Foundation Trust, Bradford Royal Infirmary, Duckworth Lane, Bradford, BD9 6RJ, UK; 2 Faculties of Life Sciences and Health Studies, University of Bradford, Richmond Road, Bradford, BD7 1DP, UK; 3 Leeds Clinical Trials Research Unit, University of Leeds, Leeds, LS2 9JT, UK; 4 Department of Health Sciences, University of York, Seebohm Rowntree Building, University of York, Heslington, York, YO10 5DD, UK; 5 Hull York Medical School, University of York, Heslington, YO10 5DD, UK; 6 Medical Research Council Integrative Epidemiology Unit, University of Bristol, Oakfield House, Oakfield Grove, Bristol, BS8 2BN, UK; 7 Population Health Sciences, Bristol Medical School, University of Bristol, Bristol, UK; 8 Institute of Population Health Sciences, Barts and the London School of Medicine and Dentistry, Queen Mary University of London, Yvonne Carter Building, 58 Turner Street, London, E1 2AB, UK

**Keywords:** COVID-19, coronavirus, children, family, mental health, health inequality, ethnicity, social determinants of health

## Abstract

The UK COVID-19 lockdown has included restricting social movement and interaction to slow the spread of disease and reduce demand on NHS acute services. It is likely that the impacts of restrictions will hit the least advantaged disproportionately and will worsen existing structural inequalities amongst deprived and ethnic minority groups.

The aim of this study is to deliver rapid intelligence to enable an effective COVID-19 response, including co-production of interventions, that address key issues in the City of Bradford, UK, and nationally. In the longer term we aim to understand the impacts of the response on health trajectories and inequalities in these.

In this paper we describe our approach and protocol. We plan an adaptive longitudinal mixed methods approach embedded with Born in Bradford (BiB) birth cohorts which have rich existing data (including questionnaire, routine health and biobank). All work packages (WP) interact and are ongoing. WP1 uses co-production and engagement methods with communities, decision-makers and researchers to continuously set (changing) research priorities and will, longer-term, co-produce interventions to aid the City’s recovery. In WP2 repeated quantitative surveys will be administered during lockdown (April-June 2020), with three repeat surveys until 12 months post-lockdown with an ethnically diverse pool of BiB participants (parents, children aged 9-13 years, pregnant women: total sample pool N=7,652, N=5,154, N=1,800). A range of health, social, economic and education outcomes will be assessed. In WP3 priority topics identified in WP1 and WP2 will be explored qualitatively. Initial priority topics include children’s mental wellbeing, health beliefs and the peri/post-natal period. Feedback loops will ensure findings are fed directly to decision-makers and communities (via WP1) to enable co-production of acceptable interventions and identify future priority topic areas. Findings will be used to aid development of local and national policy to support recovery from the pandemic and minimise health inequalities.

## Introduction

The UK, alongside countries throughout the world, is facing an unprecedented national emergency due to the rapid spread of the COVID-19 virus. Ethnic minority groups, and those living in deprived areas are bearing the brunt of the virus with increased mortality rates as a result of the disease compared with more affluent and White British populations ^1^. The increase in mortality in ethnic minority groups is likely to be due to a complex interplay of existing health co-morbidities and the pernicious social determinants of health including deprivation and poverty, which are more prevalent in these groups. The immediate response to the threat of the virus has been a stringent lockdown (implemented on 23 ^^rd^^ March 2020), effectively limiting people to their homes, followed by ongoing restrictions on daily life. As a result of the lockdown measures, schools have closed and many businesses have been unable to trade, resulting in high numbers of employed staff being ‘furloughed’, with other small businesses or self-employed workers unable to generate an income for prolonged periods. In the second half of March 2020, the Department for Work and Pensions recorded 950,000 new Universal Credit claims, which is a significant increase, and suggests unemployment rose sharply even before more stringent lockdown restrictions were introduced ^2^.

Whilst the lockdown measures have been successful in reducing the spread of the virus, there is a growing recognition of the wider impact of the COVID-19 response on vulnerable populations. Likely impacts from the restrictions imposed on these populations to limit the spread of COVID-19 may include worsening physical and mental health, a lack of access to health and other services, and economic insecurities including financial, food, housing and employment insecurities. The potential for increasing health inequalities is significant. Once the initial pandemic is under control, attention must turn to how to support vulnerable communities to emerge from this crisis and ameliorate the detrimental impacts on health, wellbeing and economic security.

The recovery from the COVID-19 pandemic will require intelligence on the health, social and economic impacts on vulnerable populations to be made available quickly to key policy and decision makers so that they can develop and implement policies and interventions to mitigate against potential longer term impacts of the COVID-19 pandemic. As budgets will be limited, it is likely that implementation of ‘recovery’ strategies will need to be prioritised to those in greatest need.

In order to make decisions about which policies to implement and when, it is vital that decision makers have access to information not only on the likelihood and severity of potential impacts, but also on the receptiveness and capacity of communities to engage with and benefit from policy interventions. Lived experiences and in-depth qualitative research will be key to knowing how best to help those who are most affected and most in need.

The Born in Bradford (BiB) research programme is in a unique position to be able to study the impact of the COVID-19 response on a key vulnerable population: pregnant women and families with pre-school, primary and/or secondary school aged children living in a highly deprived and ethnically diverse city. BiB has been following the health and wellbeing of over 36,000 Bradford residents since 2007. It hosts three birth cohort studies ^3– 5^ (see Table 1) as well as an internationally recognised programme of applied health research with a focus on health inequalities in deprived and ethnic minority populations.

**Table 1.** Description of Born in Bradford research infrastructure.

Cohort	Description	Number	Questionnaire data	Routine Data (health and education)	Recent data collection? (prior to March 2020)
Born in Bradford Family Cohort Study ^3, 6^	A prospective birth cohort which is tracking the health and wellbeing of over 13,500 children, and their parents, born at Bradford Royal Infirmary between March 2007 and January 2011. The health of these children is being tracked from pregnancy through childhood and into adult life.	13,776 Children 12,453 Mothers 3,353 Fathers	Yes- baseline and multiple time points on sub-samples from 6 months to 11 years	Yes	Yes – ‘Growing Up’ study follow-up collected between 2017–2020: N~5000 parents; N~7500 children aged between 6–11 ^a^.
Born in Bradford’s Better Start ^4^	Experimental birth cohort study in three deprived, multi-ethnic wards within Bradford. Currently recruiting.	Target N=5000 Current N~2900 ^b^	Yes – baseline and one follow-up (to date). Others are planned.	Yes	Yes –recruitment of pregnant women at routine pregnancy clinic (~26–28 weeks gestation) Also follow up ~N=600 collected Summer 2019 (infants aged between 1–3 years)
BiB 4 All ^5^	Birth cohort focusing on routine data linkage for research purposes. Currently recruiting.	Current N~2000 ^b^	No	Yes	Routine information only

Notes: ^a^ This planned follow-up had to be stopped at the start of lockdown and has not been able to restart yet; ^b^ Recruitment ongoing daily, figures rounded to nearest 100 as of 31st May 2020.

Participants in all BiB cohorts have consented to the use of their routine health and education data and to be contacted for future research. Recent recruitment ^4, 5^ and follow-ups of our cohort participants ^6, 7^ means that we have a detailed understanding of the physical and mental health, social, and economic circumstances of our families since index pregnancies/births, including data collected in the recent ‘pre-pandemic’ and ‘pre-lockdown’ period (2016-March 2020). The wealth of existing data can provide details on how life-course environmental, social and biological factors influence resilience and adverse responses to COVID-19 and its management. The recent pre-pandemic data can act as an “immediately pre-COVID baseline” to explore how the pandemic response will influence a range of outcomes in the short, medium and longer term. We also have the opportunity to follow our participants prospectively throughout the COVID-19 crisis to understand the impact of the crisis on health and well-being trajectories through this unpredictable time.

### Aim and objectives

Our aim is to rapidly collect key information across a range of BiB research infrastructure platforms to provide information in the short term to support policy and decision makers to deliver an effective COVID-19 urgent response in the City of Bradford, and nationally, and in the longer term to better understand the wider societal impacts of the COVID-19 response on health trajectories and inequalities in these.

Our objectives are to:

1. Work with stakeholders, communities and researchers to identify key issues of concern, research priorities, key topics and knowledge gaps to ensure our research addresses key issues to help plan the City’s recovery to COVID-19.

2. Collect quantitative information with BiB cohort participants to identify the health, social, education and economic impacts of the COVID-19 response for vulnerable families.

3.Collect qualitative data over time from cohort participants and other Bradford communities to explore in more detail issues related to the impact of COVID-19 response.

4. Feedback emerging findings to inform the local and national response, and adapt research methods as required in response to changing contexts and priorities.

We plan to address a range of research questions over the short term (6 months), medium term (6-12 months) and longer term (12 months onwards). We have provided illustrations of the type of research questions we will be able to answer in Table 2, but in line with our adaptive methods, these may be modified or expanded dependent on community and stakeholder priorities, changes in the virus infection rate and response to this (including subsequent local or national epidemics and further local/national lockdowns) and the changing context as our research progresses.

**Table 2.** Illustrative research questions.

Time frame	Research questions
Shorter term (6 months post lock-down)	• What behaviour changes are people making to their daily lives during the COVID-19 response, and how are they coping with these changes? • What is the impact of the COVID-19 response on families’ physical and mental health? • What is the impact of the COVID-19 response on families’ economic (e.g. financial, food, housing and employment) security. • What is the impact of the COVID-19 response on families’ access to key services (e.g. health, social care, education) • Are some groups of families (e.g. those living in deprived area, ethnic minority groups, key workers) at greater risk of experiencing short-term negative effects from the COVID-19 response? How might these negative effects be mitigated? • Are there any benefits of the COVID-19 response for different groups of families?
Medium term (12 months post lock-down)	• What is the impact of the Government’s response to the COVID-19 pandemic on the physical and mental health, wellbeing, economic security (financial, food and employment) on, and access to key services by, families living in Bradford? • Are some families (e.g. ethnic minority groups, deprived, those with insecure/low income jobs) at greater risk of negative impacts from the pandemic? • Are there protective factors (e.g. social support, job security) that make some families more resilient to the impacts? • What should the priorities of policy and decision makers be to reduce the impacts of the COVID-19 response on vulnerable families now, and in future epidemics?
Longer term (2–3 years post lock-down)	• What are the longer term impacts of the COVID-19 response on health, social, education and economic outcomes? • Are there inequalities in the longer-term recovery of families?

## Methods

### Setting

With a population of over 530,000, Bradford is the fifth largest metropolitan district in England ^8^. It is an ethnically diverse and young city situated in the North of England. Almost half of the births in the city are to women of South Asian (mostly of Pakistani heritage) and there are an increasing number of families in the city from Central and Eastern European backgrounds ^4^. Almost one-third of the city’s population is aged under 20 ^8^.

Bradford faces some important challenges, making its population vulnerable to the wider impacts of the COVID-19 and its management response. It has some of the highest levels of poverty and ill-health in England. It has an accelerating prevalence of diabetes and cardiovascular disease ^9^, due in part to its large South Asian population who are most at risk of these diseases. Almost a quarter of Bradford children live in poverty and 24% are obese at age 10/11 ^10^. There are specific structural characteristics in Bradford that make the community especially vulnerable to COVID-19, for example, a large proportion of households are classed as overcrowded ^11^. 

In England during emergencies, multi-agency groups, comprising of senior officers from organisations such as the emergency services, local authorities, NHS and community and voluntary sectors come together to co-ordinate the immediate response to and recovery from an emergency ^12^. In Bradford District, a Bradford District Gold group was established in response to the COVID-19 emergency following Government guidance. This is a group of senior officers from organisations across the District including emergency services, Local Authority, NHS, and community and voluntary sector which is coordinating the District response and recovery to COVID-19.

To support Bradford District Gold a COVID-19 Scientific Advisory Group (C-SAG) has been established to harness the research expertise and infrastructure of Bradford Institute for Health Research (BIHR) (including Born in Bradford), NHS and Local Authority partners to support Bradford District Gold. Bradford District C-SAG operates in two forms, bringing together health and business intelligence, commissioning, public health, policy and health research expertise in a multi-agency C-SAG and researcher expertise, including from Born in Bradford, in a BIHR C-SAG.

The C-SAG also benefits from the recently formed UK Prevention Research Partnership ActEarly consortium ^13^. Working in close partnership with Born in Bradford, ActEarly focusses on early life changes to improve the health and opportunities for children living in two contrasting areas with high levels of child poverty; Bradford, West Yorkshire and Tower Hamlets, London. In each of these areas, ActEarly is working with local communities, local authorities and other national organisations to understand how we can help families’ live healthier lives, with a particular focus on delivering system level change. Crucially, the consortium formally brings together decision makers across health and education with researchers and communities. This existing forum provides a platform for early implementation of research findings and recommendations into practice.

Both the BiB research programme and ActEarly use their findings to develop new and practical ways to work with families and health professionals to improve the health and wellbeing of vulnerable populations. We work in close partnership with city, regional and national policy and decision makers in health, education, environment and social care. Together, we are a ‘people powered’ research programme using engagement, co-production and dissemination to ensure communities and stakeholders have a major voice in determining our research priorities.

### Study design

In order to achieve our aims and objectives we plan, and have started, an adaptive longitudinal approach using a mixed methods convergent triangulation design. The study comprises four inter-linked work packages (WP) running in parallel, including: WP1) ongoing community consultations and co-production with key stakeholders (communities, community and voluntary sector organisations, decision makers, health and education professionals) and existing BiB research community groups; WP2) repeated longitudinal quantitative data collection with BiB families enrolled in our key birth cohorts (see Table 1); WP3) detailed longitudinal qualitative data collection with population sub-groups; and WP4) a feedback loop to ‘flex’ future research priorities according to community and stakeholder priorities.

**Figure 1.**
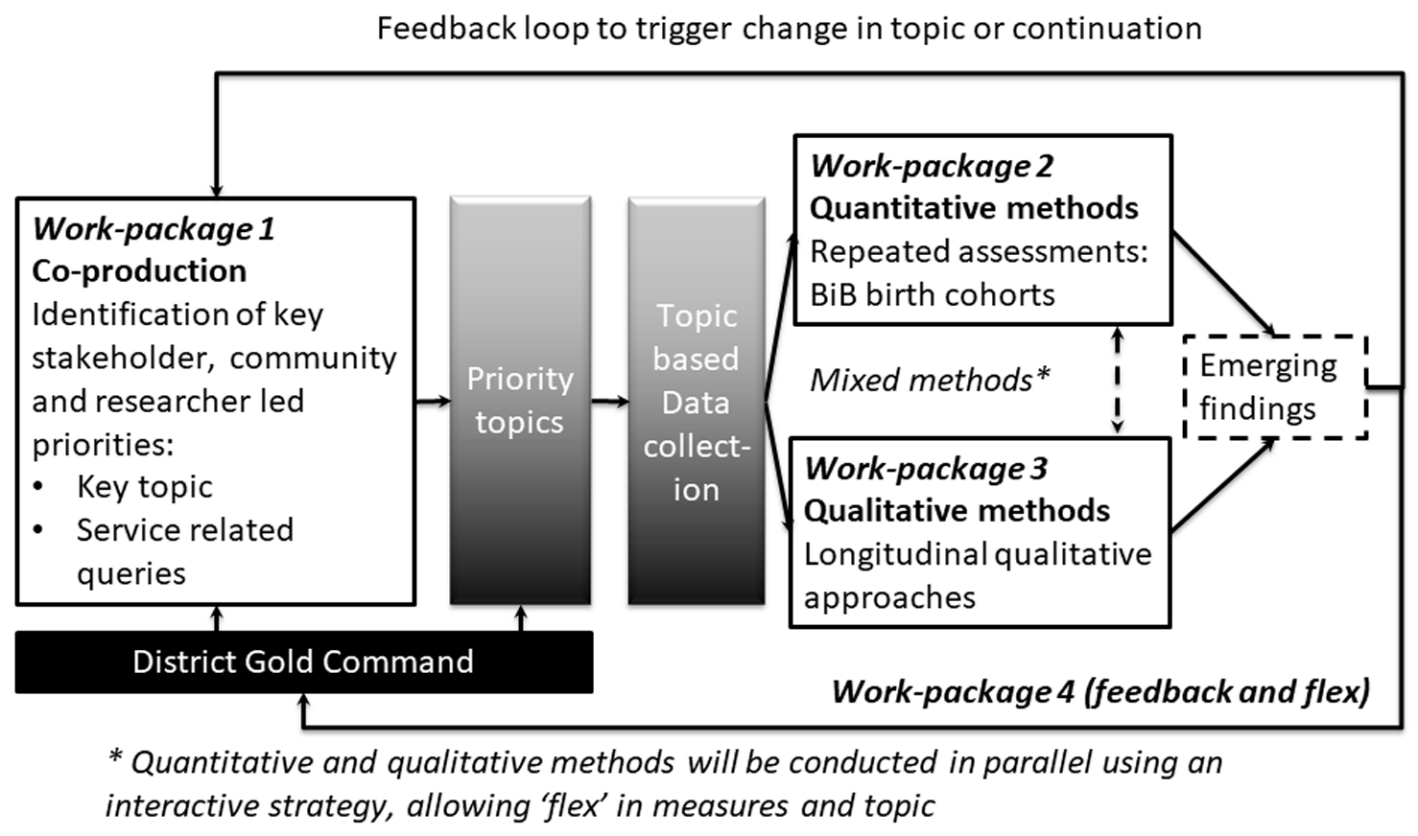
Overview of planned adaptive research methodology.

The Bradford District Gold group and national bodies (e.g. Department for Education, Public Health England, Royal College of Midwifery) will have a direct influence in setting our research focus, interpreting and disseminating findings (See Figure 1).

### Objective 1/work-package 1: co-production and engagement

Co-production of research priorities with communities and decision makers underpins the entirety of our adaptive research activities. Our approach to co-production is based on our Act Early ‘city collaboratory’ approach ^13^, and will be used in the short term to identify key research priorities and knowledge gaps, and in the longer term to co-produce interventions and initiatives to mitigate poor outcomes and health inequalities. In order to develop acceptable and feasible initiatives that have the best chance of success, we need to ensure equality and engagement of communities, stakeholders/decision makers and researchers.

Genuine co-production is predicated on reciprocal and trusting relationships between communities and other key stakeholders, ongoing dialogue, joint ownership of decision making, sharing of power and continuous reflection. It is therefore not a short term process, but one that takes time to build. As such, it is impossible to outline exactly how the co-production process will work at the outset of a programme of work. We will convene a multi-disciplinary community led steering group (including citizens, community and voluntary sector organisations, and service providers) to help us set our initial research priorities and design later stages of our research programme.

Throughout the research programme we will also seek to use varied methods of engagement and consultation (also termed ‘soft intelligence gathering’) to collect views and lived experiences of key community groups and seldom-heard communities to ensure a broad range of community perspectives are taken into consideration in the identification of research priorities and co-production activities. Our community engagement research team (SI, AR) are experienced researchers and Bradford residents who have spent many years developing genuine and trusted relationships with local community and voluntary sector organisations. We will use these links to create a direct channel of communication to discuss emerging issues, concerns and community priorities using a range of communication platforms (e.g. email, phone, text, Twitter, Facebook, WhatsApp, local media). We illustrate one example of this approach in relation to identifying immediate community concerns related to the COVID-19 lockdown in Box 1.

**Box 1. Engagement in Action – Soft intelligence gathering to explore issues experienced as a result of the lock down in key vulnerable or seldom heard communities in Bradford**

**Methods:** Informal telephone interviews with 13 key community leaders (for example, religious leaders, voluntary sector organisations, local councillors) covering a range of deprived communities within Bradford. Interviews focused on what main issues arising from ‘lock-down’ restrictions will be in short, medium and longer term. Key community groups represented included White British, South Asian, Eastern European Roma community, and Refugee and Asylum seekers. We were interested in exploring differences amongst communities

**Headline findings:**

Lock-down rules and accessing information:

→ Families living in multi-generational households find it difficult to stick to social distancing rules.

→ Awareness of rules for social distancing amongst some Eastern European Roma groups is low, in part due to low literacy levels.

→ Hoaxes and fake news regarding COVID-19 are spreading via social media channels which are causing anxiety and worry, particularly amongst South Asian Families.

Exacerbation of existing financial insecurity and poverty:

→ There were concerns across all groups of the impact of reduction in income, particularly amongst self-employed and small businesses. People reported problems in accessing financial support packages from the government.

→ Many people in Eastern European and Roma communities have ‘cash-in-hand’ jobs or agency work, and are not eligible for benefits. They may fall through the cracks in terms of receiving support

→ Food poverty was an issue particularly for larger families. For other families who need to access foodbanks, ‘essential’ items (e.g. sanitary products, soap, toothpaste) were not always available in food parcels. Families were not always able to access free school meals for children.

Accessing services, including those tackling food insecurity:

→ Families not using services which may be available (e.g. food banks), due to stigma, and / or difficulties of referral system

→ There is reduced capacity of voluntary and community sector organisations to deliver services as many are reliant on volunteers who are now not able to help due to lock-down restrictions.

Mental health

→ The impact of the COVID-19 pandemic on mental health was felt to be an important issue both in the short-term and longer term.

→ This impact is caused directly by worry and anxiety about the virus, and also indirectly by impact on financial security.

→ Face to face access to organizations for support with welfare and housing has been curtailed and this is posing a particular problem for refugee and asylum seekers.

→ Loss and grief of loved ones and friends has had an impact too as lockdown has interrupted the usual grieving process of attending funeral/burials and the mourning period.

Home and learning environment

→ For families with children, parents are struggling to access learning materials, particularly on line (which is affecting children’s education) and struggling to keep children occupied.

→ Many families do not have reliable internet access or are not able to keep a phone in credit.

Addiction

→ Individuals who have problems with addiction, who may have previously resorted to criminal means to pay for their addiction via shop-lifting or other petty crimes and can no longer do so, may turn to more extreme methods if not given help.

**How these findings have been used:** Key findings have been fed back to District Gold and are being used to inform the next phase of living with COVID-19 and laying the foundations for scenario planning for a better future for the District ^14^. They have been used to develop survey instruments and more detailed qualitative protocols to explore some of these issues in more detail (see below). We have also shared findings with local voluntary sector organisations who have reported quickly flexing provision of services in response to key issues, for example, provision of laptops for children of families in greatest need to assist education at home, and provision of ‘essential’ sanitary and hygiene products in food parcels. We have also shared findings back to participants. One participant shared the following comment: *“Thank you for getting in touch we were feeling that our needs were getting ignored until you gave us a voice. We will be happy to help again”*

See 15 for a full copy of the report.

We will harness our established research advisory groups including BiB Parent Governors (BiB Parents with children aged 9–13), BiB Young Ambassadors (BiB Children aged 9–13) and our Community Research Advisory Group (BiBBS parents with children aged 0–5). We will also use emerging findings from our quantitative research arm. For example, free text questions embedded within our large-scale quantitative surveys will ask communities about their key worries or concerns, allowing us to collect a representative sense of feeling amongst Bradford families in a way not possible by closed response questions.

Stakeholder/decision maker views and priorities will be shared via a parallel multi-agency COVID-19 scientific advisory group (including Local Authority, NHS Commissioner and Provider representatives, chaired by CC) and the Bradford District Gold (of which JW is a member). When necessary, direct input by key members will be arranged. Finally, research priorities will be shared via the BIHR C-SAG group (described above).

Our community steering group will consider collective views and priorities from all groups and together with the research team will use these to shape the direction and content of future research plans, including both quantitative and qualitative research elements. In this way we will ensure that we reflect local community needs, and provide information to decision makers than can be acted upon. Our experience to date has shown that whilst many priorities may be similar across the diverse communities within Bradford, stakeholders, and researchers, there are certain unique issues which are particularly pertinent to seldom heard or under-served communities. Without systematic exploration in a targeted way to understand the nuances in views and differences in response to the COVID-19 lockdown presented by diverse groups, there is a risk that these points may not get the consideration they deserve at decision making forums, which can potentially further widen health inequalities.

We will work with our communities, stakeholders and decision makers to jointly interpret our emerging findings, and ensure that they are disseminated in meaningful and sensitive ways. Researchers will discuss findings with members of Bradford District Gold, the multi-agency C-SAG and citizens to provide additional perspectives to support interpretation and to collectively identify final recommendations for local action in conjunction with our community steering group. Our aspiration in the longer term is to support Bradford District Gold in the development and evaluation of initiatives and interventions to mitigate against worsening outcomes and inequalities with subsequent waves and repeat epidemics, and to aid the city recovery from COVID-19 by ensuring genuine co-production with communities and stakeholders.

### Objective 2/work-package 2: quantitative surveys of the impact of covid-19 response

The main aim of this quantitative arm of our adaptive research protocol is to understand the wider impact of the COVID-19 Government response on vulnerable families using the Born in Bradford birth cohort research infrastructure (BiB, BiBBS, BiB4All, see Table 1).

***Population.*** Our sample will be drawn from the participants in our existing birth cohorts who have engaged in recent data collection to enable us to build on immediate pre-COVID-19 data (as well as other existing data from index pregnancies/birth): BiB Growing Up (BiBGU, follow-up data collection wave), data collected 2017–2020, Born in Bradford’s Better Start (BiBBS) 2016–2020 and the Born in Bradford routine data cohort (BiB4All) 2018–2020, only routine health data available for baseline). All participants in these three studies will be invited to take part in one of three key surveys:

- Sample 1: Parents – Parents of children aged 9–13 years in BiBGU and parents of children aged 0–5 years in BiBBS (total sample pool: N=7,652)
- Sample 2: Children - Children of parents in the above BiBGU sample aged 9–13 years (N=5,154)
- Sample 3: Perinatal -Women in the perinatal period (pregnancy and up to 12 months post-partum) in BiBBS and BiB4All (N=1,800)

For samples 1 and 2, surveys are planned at four time points over a one-year period with an immediate lockdown survey (April-June 2020) recently completed, and follow-up in September 2020, January 2021 and April 2021 planned.

For sample 3, pregnant women will be recruited throughout a 12 month period (June 2020-May 2021) with ongoing follow-up planned at 3, 6 and 9 months post-partum. In June-July 2020 a sub-sample of women who gave birth during the lockdown period (April-June 2020) will be recruited at the 3 months post-partum time point and followed up at 6 and 9 months post-partum.

The exact timing of the follow-up data collection periods will be flexed in response to the changing COVID-19 situation and research priorities emerging from WP1.

***Eligibility criteria***

Inclusion:

- Participant has been recruited to one of the above cohort studies and has consented to be followed-up for future research.
- For data collection via phone calls: participant is able to speak English or language also spoken by some members of the research team (e.g. Urdu/Mirpuri; Punjabi; Hungarian; Romanian).

Exclusion:

Any participant who has:

- Withdrawn from the study;
- Moved out of the Bradford District area;
- Miscarried (BiBBS/BiB4All), had a still birth (BiBBS/BiB4All), or child death (BiBGU & BiBBS & BiB4All) recorded.

***Mode of survey delivery.*** The survey for samples 1 and 3 will be completed primarily by phone with options for online and postal completion. Where participants are non-responsive by phone or email, postal questionnaires will be sent out with a stamped address envelope. Follow-ups will be conducted where postal questionnaires have not been returned within a reasonable timeframe (1-2 weeks). The survey for sample 2 will be completed by postal questionnaire addressed to the child’s parent.

***Questionnaire domains.*** Key questionnaire domains for each of the surveys used in round 1 of data collection are summarised in Table 3 below. The selected domains focus on capturing a wide range of potential impacts of the COVID-19 response across physical and mental health, living circumstances and economic, food and housing insecurity. In order to further contribute to the priority setting, open-ended questions in the survey asks about the participants’ main worries, challenges and any positive experiences as a result of the COVID-19 lockdown. Copies of the questionnaires are available on our website and as *Extended data* ^18^. We envisage that core content of the questionnaires will be repeated in each follow-up wave of data collection, but part of the nature of our adaptive research protocol is that we will be ready to ‘flex’ future waves of data collection to support collection of data on key topics identified by the first round of the survey, qualitative work and our co-production and engagement work-stream (including consideration of Bradford District Gold) as ‘priority’ areas.

**Table 3.** Key questionnaire domains.

Domain	Parent Sample	Perinatal Sample	Child Sample
Key demographics (e.g. age, ethnicity, index of multiple deprivation, socio-economic status, employment)	x	x	x
Household composition (e.g. household member clinically vulnerable to COVID-19; relationship status; housing tenure)	x	x	
Housing quality and access to outdoor space	x	x	
Insecurity of employment, finances, home & food	x	x	x
Physical health (including general health, health anxieties, health behaviours and whether self-isolated due to COVID-19)	x	x	
Mental health (including depression [PHQ-8 ^16^ and anxiety GAD-7] ^17^)	x	x	x
Family Relationships and Social Support	x	x	x
Peer support and bullying			x
Parenting competence	x		
Child behaviour			x
Loneliness & social support	x	x	
Access and use of key services	x	x	
Physical activity	x	x	x
Main worries (recorded as free text)	x	x	x
Pregnancy related health and stress		x	
Pregnancy/baby related worries and concerns and changes to perinatal care		x	
Birth plans and breastfeeding intentions		x	
Experiences of perinatal services		x	
Take up of baby immunisations		x	
The mother-child relationship (attachment)		x *	
Breastfeeding		x *	
Social support and contact with baby groups / other new mums.		x *	

Notes: *postpartum survey only.

***Analysis.*** Descriptive statistical analysis will be used to assess health, wellbeing, economic and social outcomes. Multivariable regression analyses will be used to model change from pre-COVID-19 baseline. Longitudinal trajectories of outcomes and their predictors across all survey waves will be estimated using appropriate marginal and mixed methods. All statistical analysis will be carried out using Stata 15 ^19^.

Free text questions on worries, challenges and positive experiences will be analysed using thematic analyses ^20^, employing an inductive approach where coding and theme development will be driven by the content of the responses. Codebooks will be created by a single researcher (BL) and tested by a group of researchers to test the strength and validity. Adjustments will be made, through discussion of the researchers, throughout analysis to ensure that the codebooks are reflective of the all responses.

### Objective 3/work-package 3: longitudinal qualitative methods

The main aim of the qualitative arm of this adaptive research proposal is to gain a deeper understanding of the impact of COVID-19 and the COVID-19 Government response on families in Bradford on key priority topic areas, using the Born in Bradford infrastructure as a starting point. In the medium term, the initial results and analysis of this research will support the District Gold in delivering an effective response to those families most in need. In the longer term, this information will allow a better understanding of the wider societal impacts of COVID-19 that will allow local services to prioritise their recovery of services, identify additional interventions, and inform local policy to improve resilience.

The content and focus of the initial qualitative priorities have been developed in partnership with communities and stakeholders using methods outlined in objective one. The soft intelligence gathered from communities (see Box 1) was supplemented with other sources of information:

a) Analysis of the free text responses from the first 350 parents in sample 1 of the survey on their main worries, challenges and positive experiences. In this analysis we found there was a lot of health anxiety around catching COVID-19, concerns about finances and job uncertainty, increased mental load, concerns about children’s mental health and their education as well as practical concerns such as food shopping. The analysis also found that families were enjoying spending more time together and enjoying a slower pace of life.
b) Soft intelligence gathering with members of District Gold. Brief 15–20 minute phone calls with nine members of Bradford’s District Gold to assess what their priorities were for qualitative research in Bradford in response to COVID-19. Short interviews were conducted by BL in April 2020. We first asked what they thought about the three priorities identified from our very early free text analysis of worries and concerns from our parent survey: family food security, children’s education (with a focus on children with special educational needs and disability[SEND]) and access to and experience of public/voluntary services. We then asked what would be their choice of three priorities and how they envisaged the qualitative work helping and informing their work at District Gold. These responses were recorded in note form and written up by BL. A rapid thematic analysis of the responses was conducted by BL and LS. Priorities identified were around health inequalities, poverty, domestic violence, child mental health, ethnic minority communities’ experiences and the people of Bradford’s relationship to health services as a result of COVID-19 (due to an apparent increase in mistrust and misinformation).
c) Researchers within the BIHR C-SAG identified pregnancy and the post-partum period as a potentially challenging experience during the COVID-19 pandemic. Pregnant women were identified as a group vulnerable to COVID-19 which had increased health anxieties, alongside reduced access to face to face healthcare and reduced social support due to social distancing and restricted hospital visiting.

The information on priorities received from all sources was collated and reviewed by the C-SAG group to identify the following initial priority research areas: 1) adolescent mental health, 2) health beliefs, 3) pregnancy, birth and the postnatal period, 4) impact on those families already experiencing high levels of poverty and financial insecurity.

The C-SAG agreed that detailed, longitudinal qualitative research would be particularly valuable for priorities 1 to 3 at this time. Whilst the C-SAG acknowledged the clear importance of poverty and financial insecurity, the group was aware of a recently funded mixed methods study exploring poverty in Bradford in the context of larger families which was being repurposed to address responses to the COVID-19 pandemic. For these reasons it was decided not to burden communities by instigating separate research on this priority area at this time.

Below we provide more detail on the three priority areas taken forward in the first rounds of qualitative data collection. Future topics for qualitative exploration will guided by our more formal co-production processes and our community led multi-disciplinary steering group. 

***Priority 1: Children’s mental wellbeing.*** The first of the selected priorities is children’s mental wellbeing under lockdown. Our consultations suggested that there was particular concern about secondary school age children, including the impact of social isolation, boredom, low mood and anxiety. Our definition of ‘mental wellbeing’ is broad and will be iterated as fieldwork progresses. It is important to state that we are not intending to focus the study on participants with a clinical assessment of depression or anxiety. That is, we are interested in understanding mental wellbeing concerns in the widest sense.

We are planning to conduct interviews with 20 families who have participated in both the adult and child COVID-19 surveys. The sample will be made up of two groups, the first will include children who reported moderate to low mental wellbeing in their survey and parents who raised concerns about their child(ren)’s mental wellbeing in the parent survey, and the second will include children who reported medium to high mental wellbeing in their survey and parents who did not raise concerns about their child(ren)’s mental wellbeing. We have chosen 20 in the first instance as we think this will ensure we can undertake data collection and analysis within a limited time frame whilst enabling us to obtain a diverse sample of BiB families (in terms of ethnicity, location, socio-economic background). As we are focusing on secondary school age children, these families will be from the BiBGU cohort, as the oldest children in this cohort are now aged 13 years old. BL (a post-doctoral Research Fellow with expertise in qualitative methods) will conduct two short interviews via phone/video with each family, one with a parent and the other with the child (accompanied by a parent, sibling or by themselves, whichever they prefer). The interview will focus on the child’s day-to-day life under lockdown and how they have been feeling. There will be the opportunity to do follow-up interviews post lockdown.

***Priority 2: Health beliefs.*** A priority that came through strongly in our consultation with Bradford Gold and communities was around people’s relationship to health services during the pandemic. This is a broad topic which covers changes in access to health services (and the factors affecting this), misinformation about COVID-19 spreading (especially hoax health information via WhatsApp), patients being scared to attend hospital, mistrust of health services currently, the heavy impact of COVID-19 on ethnic minority communities and bereavement/grief.

For this work, we will be sampling a range of communities in Bradford but with a particular focus on the South Asian population who seem to be more adversely affected by the above. We will conduct interviews with community leaders/trusted individuals embedded within specific communities as a starting point, then using theoretically driven snowball sampling to focus on the most affected groups. We estimate that we will conduct around 15–25 interviews but this will be determined by our assessment of data saturation. The interviews will mainly be conducted by BL except for interviews in Punjabi/Urdu where they will be carried out by other experienced qualitative researchers with these language skills.

***Priority 3: Pregnancy, birth and the post-natal period.*** This priority area will explore how the COVID-19 situation has impacted on women and partner’s experiences of pregnancy, birth and the postnatal period. A longitudinal method will be employed with interviews conducted by experienced qualitative research fellows during pregnancy, 3, 6 and 9 months post-partum. Interviews will be semi-structured with women and their partners being asked to talk about the issues that have been most important to them and/or that they are most concerned about. If they are not covered by the participant, questions and prompts that relate to the domains in the quantitative survey will be asked (see Table 3). These will be used flexibly to fit with the flow of the interview. A sub-sample of 20–30 women participating in the quantitative COVID-19 pregnancy survey will be recruited. Purposive sampling will ensure a balance of women from Pakistani and White British backgrounds and a balance from the BiBBS and BiB4All birth cohorts. In addition, 10–15 partners of the women participants will be recruited.

***Analysis.*** For priorities 1 and 2, the process of analysis will be on-going throughout recruitment and interviews and will be recorded in a research diary. The interview transcriptions will be coded manually by the lead researchers (BL, LS) independently of each other at first to ensure validity. They will take a thematic, inductive approach which will involve multiple readings of the transcripts and exploration of participant’s meanings, keeping in mind the research objectives and the survey findings. The researchers will then come together to compare and discuss emergent findings and resolve any differences through consensus. The codes that are developed will be categorised under preliminary themes, which will be ordered into themes and sub-themes to produce findings.

For priority 3, given the longitudinal nature of the study, and the focus on changes in experiences over time, the ‘pen portrait’ approach ^21^ will be used to analyse data gathered from each woman and partner. Further inductive thematic analysis across the pen portraits will allow the researchers to compare and contrast findings from men and women and across other sampling criteria. Analysis will be led by a researcher, with regular discussion and interpretation of emerging themes within a multi-disciplinary team comprising experts in maternal and child health as well as qualitative methodology.

### Objective 4/work-package 4: feedback and flex the research programme to key topic areas

Given the many uncertainties associated with the COVID-19 pandemic it is essential that our research remains responsive, evolving and flexible to continue to meet new priority research objectives and provide a meaningful contribution to the District and wider national response within the research framework. It is likely priority topic areas will continue to emerge, and change over the duration of the research. Our co-production and engagement work (work-package 1) will ensure that we continue to work with communities and stakeholders to identify topics that are most meaningful. Also, merging quantitative and qualitative findings, continued engagement and soft intelligence gathering will all shape future research activities, for example the content of follow-up quantitative surveys, and future qualitative work. Engagement with a broad range of stakeholders will ensure that a balance between immediate, medium and longer term concerns and research priorities is maintained.

### Dissemination plans

We will use a range of tailored strategies to maximise dissemination and accessibility of our findings. A key priority is to provide rapid evidence to decision makers. Locally, we have well established channels for communication (see also *Setting*). We will produce rapid briefing notes and reports based on emerging findings, which will be regularly updated. These will be distributed via Bradford District Gold and multi-agency C-SAG groups. Where briefing notes do not contain sensitive information or breach confidentiality they will be published on our C-SAG resources page. Further afield, we are already engaging with key stakeholders nationally (including Public Health England, Department for Education and Schools, Association of Directors of Public Health) and regionally (West Yorkshire Health and Care Partnership, and Yorkshire and Humber Applied Research Collaboration). Internationally, we will engage with the International Network for Research on Inequalities and Child Health, the International Society for Social Pediatrics, and Unicef, to feed into international comparative research, dissemination and policy-making.

For communities we will build on our established communication platforms, including regular newsletters with BiB families, social media channels (Facebook, Twitter and YouTube). We will create ‘in a nutshell’ findings and post these on our Born in Bradford website.

For academic audiences we will publish our findings in open access, peer reviewed journals.

### Ethical statement

The work described in work-package two has already been approved by the Health Research Authority and Bradford/Leeds research ethics committee (BiB Growing Up study 16/YH/0320; BiBBS study 15/YH/0455; BiB4All study 17/YH/0202). Ethical approval for work carried out in work-package three has been approved as an amendment to the BiB Growing Up study for children’s mental wellbeing, and as an amendment to BiBBS and BiB4All for the pregnancy, birth and postnatal period. Ethical approval for the health beliefs qualitative study has been submitted to the University of York Health Sciences ethics committee.

All participants will be provided with information about the study and contact details for the research team. Verbal consent will be taken for questionnaires completed over the phone. For online or postal questionnaire participants will informed that by completing the questionnaire they are providing consent to participate (referred to as implicit consent). This approach has been approved by our local ethics committee. Child questionnaires are sent to the parent of the child, return of the questionnaire will be taken as implicit consent by the parents. Respondents will be reassured that they do not need to answer any questions that they do not wish to and will be free to stop the survey at any time. For qualitative interviews, information sheets will be given to the participants, and verbal consent will be audio- recorded at the start of the interview, no data will be collected unless consent is recorded. For interviews with children, verbal consent from both them and their parent will be recorded. Due to social distancing requirements, all interviews will be done by phone or video.

It is possible that questions in the survey and in the interviews may cover sensitive and/or upsetting issues. All participants will be provided with a ‘useful contacts’ sheet including signposting to services able to give support for mental health, domestic violence, child abuse and education needs. It is also possible that these studies may uncover safeguarding concerns of the participant or their family. It is made clear in the information provided that confidentiality may be broken if the researcher is concerned about the safety of the participant or their family. In such cases the researcher follows the safeguarding policy of Bradford Teaching Hospitals NHS Foundation Trust.

### Study governance

A COVID-19 Scientific Advisory Group has been convened for the Bradford Institute for Health Research, which hosts Born in Bradford. This work will be overseen by this group, and by the BiB Executive Committee. In parallel we will regularly report progress to, and receive overview and scrutiny of our plans from, our existing established community research advisory groups.

### Data security and sharing

Quantitative data: All collected data will be pseudonymised and will be linked to existing research data for each participant. Data will be stored on secure NHS computer drives and in compliance with all data laws. This data will be added to the BiB and BiBBS data resources and shared with researchers anonymously as per existing procedures.

Qualitative data: Interviews will be audio-recorded and transcribed by an organisation with a privacy agreement in place. Names of interviewees and any other names mentioned within the interviews will be pseudonymised and other identifying information will be removed. Pseudoymised interview data will be stored on a secure server at Bradford Teaching Hospitals for research purposes and may be accessed as per existing procedures to access BiB data.

### Study status

The study is currently ongoing (commenced April 2020) and data collection is due to complete by May 2021.

## Discussion

We outline an adaptive research protocol harnessing the power of the Born in Bradford research infrastructure to provide rapid intelligence on the impact of restrictions imposed to limit the spread of COVID-19 in a city with high numbers of vulnerable, deprived multi-ethnic families. By definition, whilst earlier stages of the research programme are well-specified, later stages will be developed in close partnership with communities and decision makers using emerging findings and responding to priority topic areas in real time. This type of research relies on building trusting, and genuine partnerships between researchers, communities and decision-makers. We have spent many years in Bradford developing these close relationships and can now use our ‘City of Research’ infrastructure to help inform local and national recovery from the COVID-19 pandemic.

## Data availability

### Underlying data

No underlying data are associated with this article.

### Extended data

Harvard Dataverse: Extended data for BiB COVID19 Study Protocol: https://doi.org/10.7910/DVN/UQ3KDF ^18^.

This project contains the following extended data:

- WP2 BiB covid 19_invitation letter_v1 31.03.2020.pdf (Covering letter for BiB COVID-19 questionnaires)
- WP2 BiB-Child-Questionnaire-Round-1-May-2020.pdf (BiB COVID-19 Children's questionnaire administered during lock-down, May-June 2020)
- WP2 BiB-Covid-19-pregnancy-questionnaire_postal_v1.pdf (BiB COVID-19 Pregnancy questionnaire administered June 2020 onwards)
- WP2 BiB_BiBBS COVID-19 FamilyQuestionnaire_v1.pdf (BiB COVID-19 Family questionnaire administered during lock-down, April - June 2020)
- WP2 Telephone script for questionniares.pdf (Introductory telephone script for questionnaires completed over the phone)
- WP3 BiB Covid19_Parent Interview Guide_Child_wellbeing_V2.0.pdf (Parent interview topic guide for children's well-being qualitative interviews, work-package 3)
- WP3 Child information sheet_Child_wellbeing.pdf (Child information sheet and consent script for child well-being qualitative interviews, work-package 3)
- WP3 Parent information sheet_Child_wellbeing.pdf (Parent information sheet and consent script for child well-being qualitative interviews, work-package 3)
- WP3 Child Interview Guide_Child_wellbeing_V2.0.pdf (Child interview topic guide for child well-being qualitative interviews, work-package 3)
- WP3 COVID 19 Pregnancy Interview Info Sheet (Partners) v3 03.06.20.pdf (Pregnancy qualitative study: Information sheet for partners, work-package 3)
- WP3 COVID 19 Pregnancy Interview Info Sheet Mothers v3 03.06.20.pdf (Pregnancy qualitative study: Information sheet for Mothers, work-package 3)
- WP3 Pregnancy Partners_topic guide_covid study_V1.0.pdf (Pregnancy qualitative study: Interview topic guide for partners, work-package 3)
- WP3 Pregnancy Women_topic guide_covid study_V1.0.pdf (Pregnancy qualitative study: Interview topic guide for mothers (work-package 3)
- WP3 Health Beliefs Info Sheet and Consent Form.pdf (Information sheet and consent form for health belief qualitative interviews, work-package 3)
- WP3 Health Beliefs Topic Guide.pdf (Interview topic guide for health belief qualitative interviews, work-package 3)

Data are available under the terms of the Creative Commons Zero "No rights reserved" data waiver (CC0 1.0 Public domain dedication).
