# Supplementary material for: The Born in Bradford COVID-19 Research Study: Protocol for an adaptive mixed methods research study to gather actionable intelligence on the impact of COVID-19 on health inequalities amongst families living in Bradford
Source: Wellcome Open Res. 2020 Aug 13;5:191. [Version 1] doi: 10.12688/wellcomeopenres.16129.1 (PMC7523536; doi:10.12688/wellcomeopenres.16129.1)
Supplement: Peer review history

# Peer review: Reviewer response for version 1

**DOI:** 10.21956/wellcomeopenres.17704.r39953

**Published:** 2020-10-30

**Authors:** Melissa Wake

**Affiliations:** 1 Murdoch Children's Research Institute, Melbourne, Vic, Australia

Thank you for the opportunity to review the Born in Bradford COVID-19 Research Study. This will comprise a unique resource regarding the evolving impacts of the COVID-19 pandemic on children and families in a relatively disadvantaged, multi-ethnic British city during this extraordinary period. What is especially compelling is that, due to their rolling recruitment over many years, the three cohorts combined span all of childhood plus pregnancy. While I am aware of many other longitudinal child cohorts pressed into COVID service, most can report on only the narrow age band reflecting their birth window - when COVID-19 impacts are likely to differ profoundly by a child's and family's age and stage. 

 The methods are sound and appropriate, and the mixed methods and city-wide collaboration will provide a richness, breadth and depth not otherwise achievable. The speed of response in mounting this is admirable.

 I would have liked to see a greater emphasis on teasing out any positives of the pandemic. While recognising that the methods appropriately reflect the initial consultations, potential benefits may not have been uppermost in mind so could be missed by this adaptive methodology. These may include reduced preterm birth, lower rates of other infections and asthma, less school stress, more family time (and more time in general), community connectedness and clearer air. 

 I also wondered why children (unlike parents) are not asked about their own physical health or loneliness?    

 Out of interest, I wondered how the authors will balance the need to feed back findings in almost real time to the community with academic publication? 

 It will be interesting to see how the high frequency of questionnaires (multiple times in a single year) play out in short and long term response rates. Our own family focus groups here in Australia have indicated 'COVID fatigue' and a strong wish to think about something (anything!) else - especially about better times to come.  

 A few minor typos (eg 'pseudomysed' at bottom of p12) and punctuation inconsistencies could be tidied up.

 Congratulations to the BiB team on an impressive response and protocol.

Is the study design appropriate for the research question?

Yes

Is the rationale for, and objectives of, the study clearly described?

Yes

Are sufficient details of the methods provided to allow replication by others?

Yes

Are the datasets clearly presented in a useable and accessible format?

Not applicable

Reviewer Expertise:

Population child health

I confirm that I have read this submission and believe that I have an appropriate level of expertise to confirm that it is of an acceptable scientific standard.

# Peer review: Reviewer response for version 1

**DOI:** 10.21956/wellcomeopenres.17704.r40154

**Published:** 2020-09-28

**Authors:** Ingrid Wolfe

**Affiliations:** 1 Department of Women's and Children's Health, School of Life Course Sciences, Faculty of Life Sciences and Medicine, King's College London, London, UK

Thank you for asking me to review the Born in Bradford COVID-19 Research Study protocol for an adaptive mixed methods research study to gather actionable intelligence on the impact of COVID-19 on health inequalities among families living in Bradford. Reading this review was an enjoyable and educational experience, and as very occasionally happens, I am left inspired and more informed by conducting this review. 

 The Bradford Institute and Act Early initiative are well positioned to deliver the plans set out in this protocol. Actionable practical research, co-designed and co-produced, delivering rapid intelligence to decision-makers, and within an agile adaptive research design that allows iterative learning and revision is an example for us all to follow in such challenging times. 

 The rationale and methods are clearly set out, and could – indeed should – be reproduced elsewhere. The research design comprises co-production of research priorities, followed by mixed methods research with appropriate analytic methods, and finally a nicely-thought through feedback loop for co-design of interventions and/or further iterations of research. The four work packages set out in a logic model that allows continuous feedback and adaptation is well thought through and nicely designed both to speak to decision makers to support them in policy-making, and to inform research priorities for longer term work. 

 My comments and suggestions are minor. The introduction section describes the interaction between the COVID-19 pandemic and other factors – comorbidities, ethnicity, and social determinants. These interdependencies can be described as syndemic, which could provide a useful perspective in analysis (see for example Horton R. Offline: COVID-19 is not a pandemic DOI ^1^). My only other comment is that the one of the three initial priority research areas is described as *adolescent* mental health in one area (page 10, penultimate paragraph), and *children’s* mental wellbeing in another (page 11 second para). The former seems right, given that the sampling is from secondary schools; it may seem quibbling to point this out, but to a purist child health researcher it might stick out. 

 I commend this protocol without hesitation, am confident that it will deliver actionable findings to local decision makers, and probably national ones too. I look forward to reading the results.

Is the study design appropriate for the research question?

Yes

Is the rationale for, and objectives of, the study clearly described?

Yes

Are sufficient details of the methods provided to allow replication by others?

Yes

Are the datasets clearly presented in a useable and accessible format?

Yes

Reviewer Expertise:

Child health systems and policy research; paediatrics.

I confirm that I have read this submission and believe that I have an appropriate level of expertise to confirm that it is of an acceptable scientific standard.

## References

[1] : Offline: COVID-19 is not a pandemic. *The Lancet*.2020;396(10255) : 10.1016/S0140-6736(20)32000-6 10.1016/S0140-6736(20)32000-6 PMC751556132979964

# Peer review: Reviewer response for version 1

**DOI:** 10.21956/wellcomeopenres.17704.r40153

**Published:** 2020-09-17

**Authors:** Michelle McKinley

**Affiliations:** 1 Centre for Public Health, Queen's University Belfast, Belfast, UK

The Born in Bradford (BiB) COVID-19 research study is a timely piece of research that aims to provide information in the short term to support policy and decision makers to deliver an effective COVID-19 urgent response in Bradford and nationally in the UK, and in the longer term will allow a better understanding of the wider societal impacts of the COVID-19 response on health trajectories and inequalities. The adaptive longitudinal mixed methods design will allow an agile response to COVID-19 developments and community and stakeholder priorities. The short, medium and longer term research questions will be addressed by four inter-linked work packages, focused around the BiB cohorts, that will run in parallel. These comprise: WP1) ongoing community consultations and co-production with key stakeholders (communities, community and voluntary sector organisations, decision makers, health and education professionals) and existing BiB research community groups; WP2) repeated longitudinal quantitative data collection with BiB families enrolled in BiB birth cohorts; WP3) detailed longitudinal qualitative data collection with population sub-groups; and WP4) a feedback loop to ‘flex’ future research priorities according to community and stakeholder priorities.

 The rationale and objectives of the study are clearly described and the methods are clear and well justified. The existing BiB research infrastructure has been built up over many years and this COVID-19 research study sits within this infrastructure and is uniquely placed to provide ‘actionable intelligence’ on health inequalities in a timely manner. This is a robust, well written and presented protocol and I have no edits to suggest apart from one minor suggestion to include some information on whether participants are given any payments/vouchers/reimbursements for their participation in the research or describe other approaches that are used to encourage participation.

Is the study design appropriate for the research question?

Yes

Is the rationale for, and objectives of, the study clearly described?

Yes

Are sufficient details of the methods provided to allow replication by others?

Yes

Are the datasets clearly presented in a useable and accessible format?

Yes

Reviewer Expertise:

Nutrition, obesity, behaviour change

I confirm that I have read this submission and believe that I have an appropriate level of expertise to confirm that it is of an acceptable scientific standard.
